# Supplementary material for: Using random forests to uncover the predictive power of distance-varying cell interactions in tumor microenvironments
Source: PLoS Comput Biol. 2024 Jun 14;20(6):e1011361. doi: 10.1371/journal.pcbi.1011361 (PMC11210873; doi:10.1371/journal.pcbi.1011361)
Supplement: S1 Text — Appendices A-E, detailing supporting details regarding edge corrections (A), surrogate splitting methods (B), as well as empirical evidence from simulations supporting choice of B and H parameters (C), investigations of summary metrics OOB, BIAS, and GUESS (D), and consideration of performance under low sample, high interaction count setting (E). (PDF) [file pcbi.1011361.s001.pdf]

S1 Text: Supporting information for “Using random forests to uncover the predictive power of distance–varying cell interactions in tumor microenvironments”

Jeremy VanderDoes[1], Claire Marceaux[2,3], Kenta Yokote[2], Marie-Liesse Asselin-Labat[2,3], Gregory Rice[1], Jack D. Hywood[4]*

1 Department of Statistics and Actuarial Science, University of Waterloo, Waterloo, Canada

2 Personalised Oncology Division, The Walter and Eliza Hall Institute of Medical Research, Parkville, Australia

3 Department of Medical Biology, The University of Melbourne, Parkville, Australia

4 Department of Anatomical Pathology, Royal Melbourne Hospital, Melbourne, Australia

A Edge corrections

It is common when computing such K functions to correct them for what are referred to as “edge effects”. Edge effects describe the issue that cells near the edge of an image appear to have fewer cells around them when r extends beyond the nearest boundary. One option is toroidal edge corrections as described in [1], which replicates and reflects the image data occurring near the boundary. Another is isotropic edge corrections which weights edge cells, see [2–4]. We examined several methods and found similar results. We use a standard isotropic edge correction in this paper. For a single point x in some image I, then the isotropic edge correction weight is

$$e(u,r) = \frac{2\pi r}{c(u,r) \cap I} \tag{1}$$

where the denominator is the intersection of the circle $c(u, r)$ with radius r and center u. For discussions on approaches to edge corrections see [5, 6].

B Surrogate splitting

The principle behind surrogate splits is that at each split a selection for the splitting variable must be made, even if the selection is between nearly identical variables. Traditional splitting would select only one, giving it a measure of variable importance, and ignore any other options. In this way, traditional splitting can result in masked variables and inaccurate representations of variable importances. A model using surrogate splits revisits each split after selecting the "primary" splitting variable and considers alternative, "secondary" splitting variables, mitigating the masking behavior.

Surrogate splits also take into account that the variables used after the primary split are, to some degree, lower quality and weight the splits accordingly. Weighting is performed according to a split's impurity reduction, and varies slightly based on classification or regression problems [7]. Moreover, these surrogates allow for use of another split if an observation is missing data for the primary split.

Consider that a naïve choice for any variable missing data used in the primary split would be to go with the majority split. In order to evaluate potential splits, they should perform better than this naïve approach. In particular secondary splits should outperform this approach on missing data, and perhaps even split nearly as good as the primary split on all data. Consequently, one potential weighting scheme for node A and potential surrogate v is

$$w_v(A) = \frac{c_v - c_{maj}}{n_A^{(\text{node})} - c_{maj}} \tag{2}$$

where c_v is the number of observations correctly classified due to splitting by variable v, c_{maj} is the number of observations correct by naïvely selecting the majority class, and $n_A^{(\text{node})}$ is the total number of observations in the node.

A typical variable may appear many times in a tree, both as a primary and surrogate splitting variable. Therefore, the overall measure of variable importance for a

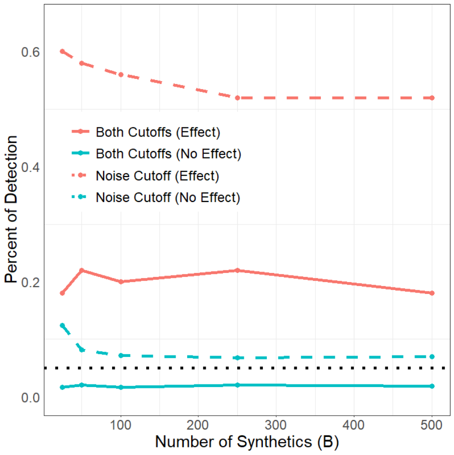

(a) 4 cell types.

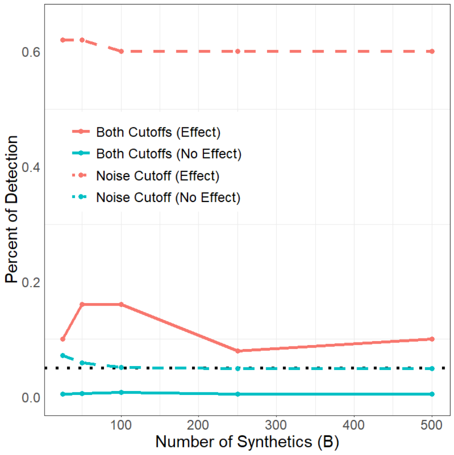

(b) 16 cell types.

Fig C1. Selection of B. Simulations of into the number of synthetics, B, for (a) 4 and (b) 16 cell scenarios. In both, only one interaction, $c2\_c2$, is significant, and all other interactions are non-significant. Per Figure 8 in the main manuscript, the expected effect is mild (standard deviation in placement of the cells from the Thomas process changing from 1/25 to 1/60). Running 50 trials for significance of 0.05, the power is reasonably level across selections of B, excluding (expected) low power when the number of synthetics is far too low to accurately approximate the desired quantile.

given variable is a combination of the splitting importances

$$\mathrm{VI}(v) = \frac{1}{|\text{Number of Trees}|}\left(\sum_{i\in P_v}\Delta I_i + \sum_{s\in S_v} w_s \Delta I_s\right) \quad (3)$$

where for variable v, P_v and S_v respectively indicate the set of primary and surrogate splits.

C Choosing B and H

The parameters B and H are user selected and are used in computing the thresholds for evaluating variable importance. The parameter B defines the number of synthetic spatial variables simulated independently of the response of interest used in the proposed procedure while parameter H defines the number of iterations for quantile estimation. This section demonstrates the effect of these choices via simulations similar to those in the section Simulation study in the main manuscript, which mimic the TNBC dataset.

Throughout these simulations, we set $H = B$. We considered both the 4 cell type and 16 cell type experiments as defined in the section Simulation study for varying

choices of B. The data were simulated so that only one interaction ($c2\_c2$) was related to the response, and all other interactions were simulated independently of the response. A mild interaction effect was imposed, with underlying standard deviation in placement of the cells changing from 1/25 to 1/60 between the binary outcomes for the modified Thomas process. The percentage of variable importance values exceeding the noise and interpolation thresholds, calibrated to the 95% level were recorded for 50 independent simulation trials. Figure C1 shows these percentages as a function of $B \in \{50, 100, 250, 500\}$ for the interaction that has a difference between groups ($c2\_c2$) and for the interactions that do not have a difference (shown as the mean of all other interactions). We observed that the two thresholds (i.e. the 95% threshold using noise only and that using both noise and the interpolation threshold) showed relative stability in terms of their false positive and false negative rates for all values of B exceeding 50. In terms of estimating the two thresholds at the 95% level, there was no apparent advantage to choosing a larger value of B for the number of cell interactions considered. Since increasing B increases the computational burden without obvious benefits we recommend $B = H = 100$ as default choices.

D OOB accuracy and naïve model comparisons

We also investigate the summary metrics OOB, BIAS, and GUESS. We conduct simulations similar to those described in the Simulation study section – we consider the case of 16 cell types in two scenarios: one in which the spatial interactions were independent of the response (null) and when they were dependent on the response (alternative). Each scenario was simulated 100 times, and we recorded the OOB value. The summary results of these simulations are given in Tables D1 and D2. In the null case, the OOB was largest among the three metrics at approximately the same rate. However, in the alternative case, OOB far exceed the other estimates in terms of value and rate being the largest.

	OOB	BIAS	GUESS
Mean	0.431	0.500	0.500
Median	0.438	0.500	0.500
Std. Deviation	0.134	0.000	0.000
Largest (%)	0.360	0.640	

Table D1. Null Summary Metrics Table. In 100 simulations without differences between the outcome groups (i.e., the null scenario), we compare the out-of-bag (OOB) accuracy of the true model to the naïve approaches of always selecting the most common outcome (BIAS) and selecting a random outcome based on frequency (GUESS)– see Predictive accuracy estimates and Simulation study sections in the main manuscript for more details. The table shows the methods are unskewed and all randomly report the largest values about an equal number of times (about one third for each metric). Compare this table to Table D2.

	OOB	BIAS	GUESS
Mean	0.845	0.500	0.500
Median	0.850	0.500	0.500
Std. Deviation	0.072	0.000	0.000
Largest (%)	1.000	0.000	

Table D2. Alternative Summary Metrics Table. In 100 simulations with a difference between the outcome groups (i.e., the "alternative" scenario), we compare the out-of-bag (OOB) accuracy of the true model to the naïve approaches of always selecting the most common outcome (BIAS) and selecting a random outcome based on frequency (GUESS)–see Predictive accuracy estimates and Simulation study sections in the main manuscript for more details. The table shows the methods are unskewed and OOB consistently reports the largest values, showing the model does capture the existing information–that is the model does seem to recognize informative interactions and/or meta-variables. Compare this table to Table D1.

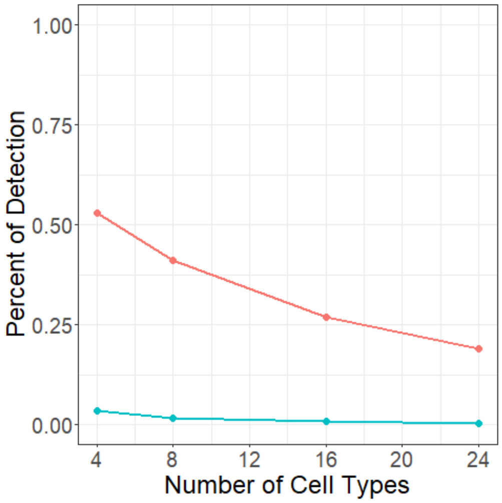

Fig E1. Mean detection rate of the significant interaction (red) and mean detection rate of all non-significant interactions (blue) in a small sample scenario with an increasing number of cell types. In each scenario, 100 iterations per scenario, all interactions were considered ($T(T+1)/2$ interactions). As the number of interactions increase, the method suffers only minor power loss while controlling for false discovery rate.

E Performance in the low sample, high interaction count scenario

It is of value to know the performance, i.e. the detection and false discovery rates, of models in sub-optimal situations. For our model, sub-optimal can be taken to indicate a small sample size or a large number of interactions.

In this section, we consider simulations for small samples of 20 patients, 10 "positive" and 10 "negative" with an increasing number of interactions. For each patient, a single image is recorded, with no meta-variables. The cells in the images are placed according the process described in Simulation study. No interactions between cell types are present and all images contain only a single, weak interaction between one cell type that differs between the positive and negative groups. The number of each cell type is random, selected via a Binomial distribution with 50% inclusion and 100 possible cells. The number of cell types, and therefore the number of interactions, is varied and each scenario is iterated 100 times. In each iteration, the variable importance, and its relation to the thresholds, is recorded to investigate the ability of funkycells to detect the significant interaction and mitigate false discovery of non-significant interactions.

The results of the simulation, showing the detection of the significant interaction (red) and the mean of all non-significant interactions (blue), is given in Figure E1. In the 4 cell type case, the significant interaction is detected only slightly over 50% of the time. Yet, the power loss is small as the number of unique cell types increases. Yet while 4 unique types corresponds to only 10 interactions, 24 unique types corresponds to 300 interactions. Even under the high interaction count scenario, the method detects the true interaction far more than any other non-significant interaction. In fact, using both thresholds the model becomes more conservative as the number of interactions is increased, managing the false discovery rate. Unreported figures considering only the noise threshold or only the interpolation threshold shows each holds detection of non-significant interactions to the specified significance level α.

References

1. Ripley BD. Tests of 'Randomness' for Spatial Point Patterns. Journal of the Royal Statistical Society Series B, Methodological. 1979;41(3):368–374.

2. Ohser J, Stoyan D. On the Second-Order and Orientation Analysis of Planar Stationary Point Processes. Biometrical journal. 1981;23(6):523–533.

3. Ripley BD. Statistical inference for spatial processes. Cambridge: Cambridge University Press; 1988.

4. Baddeley A, Rubak E, Turner R. Spatial Point Patterns: Methodology and Applications with R. London: Chapman and Hall/CRC Press; 2015. Available from: `https://www.routledge.com/Spatial-Point-Patterns-Methodology-and-Applications-with-R/Baddeley-Rubak-Turner/9781482210200/`.

5. Haase P. Spatial pattern analysis in ecology based on Ripley's K-function: introduction and methods of edge correction. Journal of vegetation science. 1995;6(4):575–582.

6. Yamada I, Rogerson PA. An Empirical Comparison of Edge Effect Correction Methods Applied to K-function Analysis. Geographical analysis. 2003;35(2):97–109.

7. Breiman L, Friedman J, Stone CJ, Olshen RA. Classification and Regression Trees. Chapman and Hall/CRC; 1984.
