# Supplementary material for: Using random forests to uncover the predictive power of distance-varying cell interactions in tumor microenvironments
Source: PLoS Comput Biol. 2024 Jun 14;20(6):e1011361. doi: 10.1371/journal.pcbi.1011361 (PMC11210873; doi:10.1371/journal.pcbi.1011361)
Supplement: S1 Table — Table summarizing the patients, number of images, type of lung cancer, and the cell count for each type. (PDF) [file pcbi.1011361.s002.pdf]

Patient (Images)	Cancer	B	CD4T	CD8T	Dendritic	Granulocytes	Macrophages	Mast	NK	Treg	ydT
1 (2)	LUAD	152	148	93	199	123	178	137	17	48	7
2 (1)	LUAD	391	248	286	57	3	144	410	17	32	4
3 (2)	LUAD	1752	699	981	75	68	278	14	51	69	46
4 (3)	LUSC	18	89	48	30	13	37	61	3	66	12
5 (1)	LUSC	6	30	32	5	19	6	22	2	19	2
6 (1)	LUAD	290	330	206	22	442	99	12	8	35	11
7 (1)	LUAD	669	509	791	122	27	129	139	9	25	18
8 (1)	LUAD	14	33	62	6	9	27	13	2	2	4
9 (1)	LUAD	9	47	83	3	2	10	16	1	3	1
10 (1)	LUAD	35	188	143	48	13	117	11	66	32	48
11 (3)	LUSC	340	394	115	77	134	186	141	19	57	21
12 (1)	LUSC	27	78	89	0	6	31	78	3	0	2
13 (2)	LUAD	16	52	96	11	27	91	43	26	64	11
14 (1)	LUSC	623	271	224	247	490	164	154	16	64	30
15 (4)	LUAD	320	215	232	2	49	89	22	13	2	34
16 (4)	LUAD	303	376	211	22	206	100	5	30	75	45
17 (1)	LUAD	443	106	45	16	33	8	12	0	8	0
18 (2)	LUAD	28	166	211	79	39	61	488	7	8	12
19 (1)	LUAD	30	57	49	12	9	43	38	40	15	2
20 (1)	LUSC	24	14	30	11	122	72	13	0	1	0
21 (1)	LUAD	474	11	24	36	13	4	0	0	1	4
22 (2)	LUAD	533	186	160	87	79	69	3	17	40	44
23 (1)	LUAD	488	282	124	81	6	172	7	9	25	13
24 (1)	LUAD	16	172	54	4	32	170	45	5	36	4
25 (3)	LUAD	216	253	169	78	1404	97	17	23	4	74
26 (5)	LUSC	7	251	190	112	748	207	68	66	113	20
27 (3)	LUSC	15	271	429	9	382	63	10	13	10	103
28 (1)	LUAD	12	72	35	1	0	9	3	0	5	3
29 (1)	LUAD	238	314	265	78	23	171	65	18	83	11
30 (1)	LUAD	11	12	15	0	2	3	24	0	2	4
31 (4)	LUAD	482	312	536	190	25	101	30	10	19	55
32 (5)	LUAD	148	690	806	623	60	842	62	19	104	62
33 (3)	LUAD	110	469	199	51	279	177	89	10	42	15
34 (7)	LUSC	694	1521	386	460	463	158	350	94	135	87
35 (4)	LUAD	1023	280	262	141	2	329	448	21	34	33

Patient (Images)	Cancer	B	CD4T	CD8T	Dendritic	Granulocytes	Macrophages	Mast	NK	Treg	ydT
36 (1)	LUAD	12	26	103	0	97	57	15	9	0	3
37 (1)	LUSC	5	4	8	0	7	4	1	0	1	0
38 (1)	LUAD	275	73	85	22	1206	110	7	2	3	11
39 (2)	LUAD	184	466	543	163	12	145	18	19	37	20
40 (4)	LUAD	9	11	29	0	197	45	5	3	8	4
41 (2)	LUSC	9	21	30	2	21	39	10	21	13	3
42 (3)	LUAD	67	300	398	41	4	369	25	5	52	39
43 (1)	LUSC	20	12	13	3	6	59	2	1	0	0
44 (1)	LUSC	19	22	15	4	3	39	5	3	5	4
45 (2)	LUAD	10	90	43	8	2	43	9	6	3	1
46 (1)	LUSC	3	21	76	0	6	44	10	0	48	3
47 (1)	LUAD	17	0	1	4	0	11	0	0	0	0
48 (2)	LUAD	699	200	165	23	1	181	35	5	25	25
49 (9)	LUAD	69	324	387	18	8	520	350	5	18	26
50 (7)	LUAD	1715	1058	869	360	765	402	58	142	103	36
51 (1)	LUSC	76	69	95	5	771	144	26	4	35	3
52 (7)	LUSC	538	745	885	23	243	518	257	20	622	64
53 (5)	LUSC	240	340	458	104	623	728	100	35	117	64
54 (6)	LUAD	70	90	292	23	4	247	122	168	78	38
55 (6)	LUAD	59	313	151	140	324	265	181	14	24	31
56 (2)	LUSC	62	176	113	17	30	248	21	7	27	17
57 (1)	LUSC	6	112	274	2	37	129	0	3	8	8
58 (1)	LUAD	62	0	0	0	9	2	0	0	0	1
59 (6)	LUAD	5	200	80	184	98	545	290	104	166	45
60 (1)	LUAD	122	33	57	14	0	90	17	4	20	2
61 (1)	LUAD	408	74	44	6	101	44	33	0	8	4
62 (1)	LUAD	2	0	0	0	0	0	0	6	0	0
63 (1)	LUAD	767	976	1409	358	37	817	43	38	245	37
64 (1)	LUSC	13	108	62	2	0	22	9	2	1	0
